# Crocin: Functional characteristics, extraction, food applications and efficacy against brain related disorders

**DOI:** 10.3389/fnut.2022.1009807

**Published:** 2022-12-13

**Authors:** Anwar Ali, Liang Yu, Safura Kousar, Waseem Khalid, Zahra Maqbool, Afifa Aziz, Muhammad Sajid Arshad, Rana Muhammad Aadil, Monica Trif, Sakhawat Riaz, Horia Shaukat, Muhammad Faisal Manzoor, Hong Qin

**Affiliations:** ^1^Xiangya School of Public Health, Central South University, Changsha, China; ^2^Department of Research and Development Office, Hunan First Normal University, Changsha, China; ^3^Department of Food Science, Government College University Faisalabad, Faisalabad, Pakistan; ^4^National Institute of Food Science and Technology, University of Agriculture, Faisalabad, Pakistan; ^5^Food Research Department, Centre for Innovative Process Engineering, Syke, Germany; ^6^Department of Home Economics, Government College University, Faisalabad, Pakistan; ^7^Food and Nutrition Society, Gilgit Baltistan, Pakistan; ^8^Guangdong Provincial Key Laboratory of Intelligent Food Manufacturing, Foshan University, Foshan, China; ^9^School of Food Science and Engineering, South China University of Technology, Guangzhou, China

**Keywords:** crocin, extraction techniques, food applications, medicinal, brain disorders

## Abstract

Crocin is a bioactive compound that naturally occurs in some medicinal plants, especially saffron and gardenia fruit. Different conventional and novel methods are used for its extraction. Due to some control conditions, recent methods such as ultrasonic extraction, supercritical fluid extraction, enzyme-associated extraction, microwave extraction, and pulsed electric field extraction are widely used because these methods give more yield and efficiency. Crocin is incorporated into different food products to make functional foods. However, it can also aid in the stability of food products. Due to its ability to protect against brain diseases, the demand for crocin has been rising in the pharmaceutical industry. It also contain antioxidant, anti-inflammatory, anticancer and antidepressant qualities. This review aims to describe crocin and its role in developing functional food, extraction, and bioavailability in various brain-related diseases. The results of the literature strongly support the importance of crocin against various diseases and its use in making different functional foods.

## Introduction

Crocins, a series of polyene dicarboxylic acid, mono and di-glucosyl esters of crocetin, are the major colors causing compounds of saffron and gardenia. In China, the contents of gardenia fruits are used as herbal remedies and natural colors (1). Other plants, such as *Buddleja spp*., also generate crocins, but because of their low concentration, they are not commercially utilized (2). Crocin is a chemical diester composed of the dicarboxylic acid crocetin and disaccharide gentiobiose (3). Crocins are crocetin glycosyl esters generated *via* the esterification of crocetin with various glycosides, including geometric isomers (3). The activity of glucosyltransferases causes the transfer of crocetin molecules, which add varying amounts of glycosidic to yield crocins, a primary component of saffron that confers water solubility (4). Crocins are responsible for many of this valuable plant's pharmacologic effects (5). Crocins, unlike other carotenoids, have 20 carbons and several glycosides. Several earlier studies demonstrated that crocins, particularly alpha crocin, had radical quenching, antioxidant, and anti-inflammatory properties (6).

Crocin (mono- or di-glycosyl polyene esters) is a key bioactive component in saffron that dissolves easily in water and produces a distinctive red color, making saffron a natural coloring compound (7). Trans-crocetin di-(-D-gentiobiosyl) ester, crocin1 is the most prevalent crocin, with a golden-yellow tint. Crocin has the highest recorded absorbance at 440 nm (8). Because of its limited stability, crocins lose its functionality when exposed to light, heat, and acidic nature (9). Also have low immersion and bioavailability, as it is un-absorbed if taken orally till hydrolyzed to produce deglycosylated trans-crocetin in the intestinal tract by enzymatic conditioning in the intestinal epithelial cells and by the fecal bacteria (9).

Crocin has several pharmacological actions, including anti-inflammatory and cancer cell growth inhibitor properties (10–12). Under various experimental settings, crocin has also been shown to protect against oxidative damage to brain vasculature, renal tissues, the heart, and the retina (3). In addition to their anti-hypertensive, anti-platelet aggregation, nephron-protective effects, anti-depressant, and anti-atherosclerotic, these phytoconstituents are radical scavengers, particularly against superoxide anions (13). Many people are afflicted with neurodegenerative disorders such as epilepsy, Parkinson's, and Alzheimer's, with increasing age being the primary risk factor (14). The naturally occurring carotenoid molecule, crocin, has been shown to offer therapeutic potential in treating neurological disorders (15). Crocin also increases dopamine in the brain during Parkinson's disease. As a result, this chemical has been demonstrated to be a promising treatment option for neurodegenerative diseases (16). According to randomized, double-blind, placebo-controlled experiments, crocin medicinal levels do not harm the body. In a double blind randomized clinical trial it was found that crocin (20 mg/day) is safe in healthy volunteers, with no notable changes in hematological, hormonal, biochemical, or urine markers (17). This review aims to describe the role of crocin in developing functional food, extraction and critically evaluate prior and current findings on the biological/pharmacological actions of crocin against various brain related diseases.

## Biochemical structure of crocin family

The crocins are a class of hydrophilic carotenoids that are either mono- or di-glycosyl polyene esters of crocetin in which D-gentiobiose and/or D-glucose appear as carbohydrate residues (18). A carotenoid with a 20-carbon dicarboxylic structure. Saffron contains a variety of carotenoid chemicals, containing trace levels of zeaxanthin, alpha and beta carotene, and lycopene (19).

Six different forms of the crocin family's glycosyl esters have been found in saffron. Trans-crocins 3 and 4 are the most prevalent of the crocin analogs, which include crocins 1–4 and are virtually glycosides of trans-crocetin in saffron. These crocins range in concentration in Spanish saffron between 0.01 and 9.44 percent and 0.46 and 12.12 percent, respectively, whereas cis-crocetin and its glycosides are minor constituents (20). Except for crocin-1, all crocin derivatives are said to exist as pairs of cis-trans isomers. Trans-crocins undergo photoisomerization events and change to cis-crocins, according to a research by (21); this process is reliant on the agro-ecological circumstances in the region of the plant's origin.

**Figure 1:**
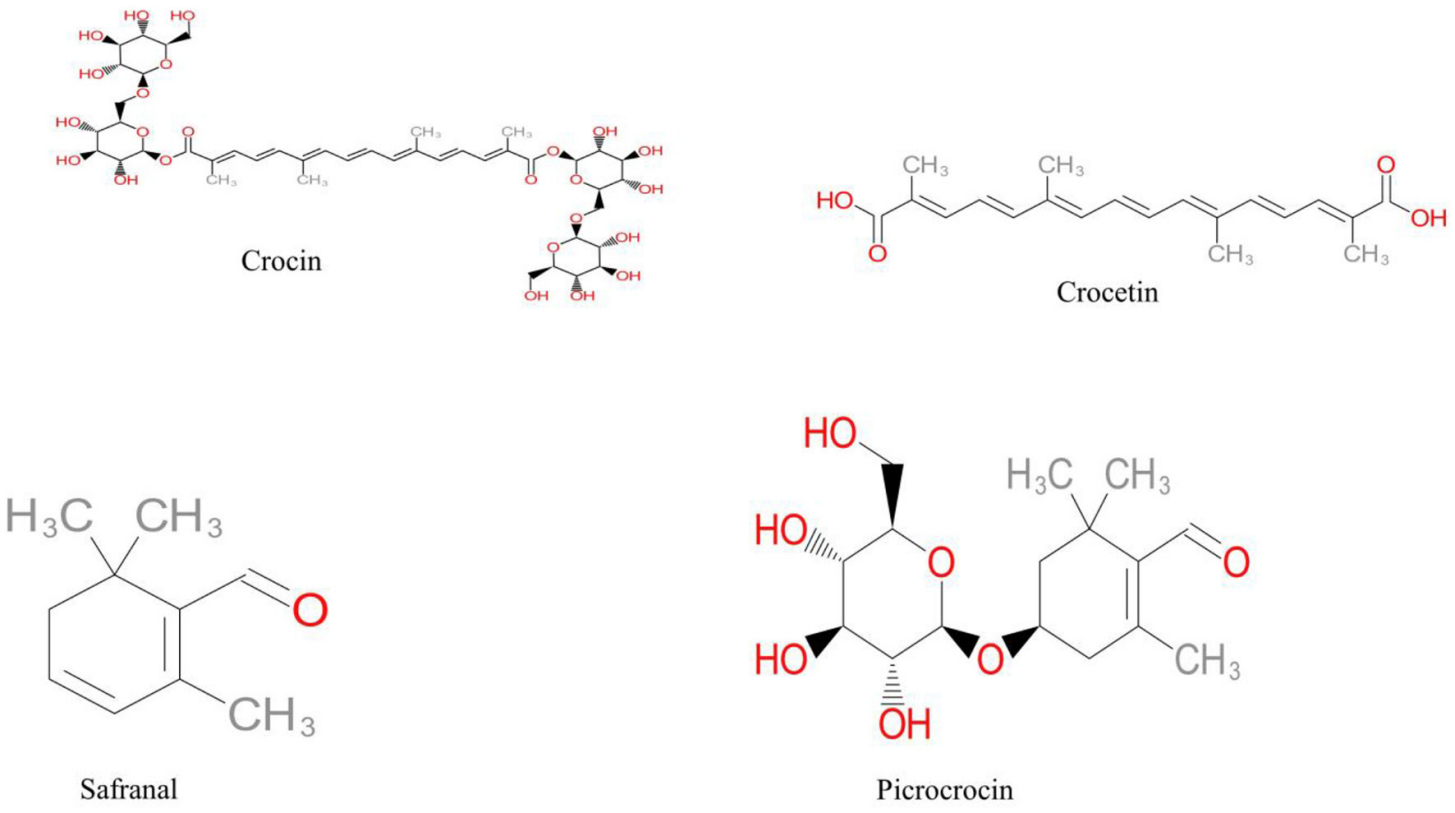
Chemical structure of crocin, dicarboxylic acid (crocetin), monoterpen aldehyde (safranal) and glycoside (picrocrocin).

Due to its high water solubility, crocin, also known as alltrans crocetin di-b-D-gentiobiosyl ester, has the best coloring capacity when compared to the other carotenoides of saffron. Crocin, which is also soluble in methanol and ethanol, is considered as the preferred water-soluble food additive because of its ability to quench free radicals and possess tumor-fighting characteristics. Structure of crocetin and its glycosides are presented in (Figure 1).

## Different plant sources of crocin

Crocin is the pigment that gives saffron its color (22). It is also taken from the fruits of the gardenia (23). It occurs as a red powder as a solid, yet it gives a yellow color to dishes (24). crocin as a spice produced by the s *Crocus Sativus* L. is produced primarily in Western Asia, with Iran being the world's largest producer, but it is also economically significant in huge sections of Mediterranean Europe (25). The bitter flavor of a saffron spice is attributed to the monoterpene glycoside picrocrocin, whereas the scent is attributed to the aglycone safranal (26). The closed flowers of saffron are hand-picked in the early morning to ensure greater resistance to the degenerative processes of the floral organs and obtain a spice with high qualitative traits (25). A mechanical system can also do harvesting with two primary parts: the first detaches the corolla from the stem, and the second gathers the removed flower using a vacuum collector (25). It takes 370–470 h to make 1 kg of dried saffron through manual plucking (27). Using high-performance liquid chromatography (HPLC), the principal features of the saffron extract related to crocins and safranal concentration were identified (28).

Gardenia is an evergreen shrub commonly used in landscaping with characteristics like sweet, softly aromatic blossoms (29). It is a well-known fruit for ayurvedic purposes in China due to its chilly and bitter characteristics (30). Therefore, medicinal importance of this shrub includes curing stomach aches and hepatic, as well as treat diuretic, anti-phlogistic, choleretic, laxative, and homeostatic qualities (30). Also, it is used to obtain yellow color since it contains crocin and crocetin, primary plant carotenoid constituents (30).

The derivatives derived from *G. jasminoides* are less poisonous, less allergenic, and more environmentally friendly than saffron (31). A homogenate extraction procedure was used to extract crocin from *G. jasminoides* (32, 33). Another work used ethanol/water cold percolation to extract crocin from *G. jasminoides* without affecting percolation (34). The Microwave-assisted extraction (MAE) method boosted the extraction yield of crocin from *G. jasminoides'* edible yellow pigment by 50% over the usual extraction method (35).

Crocin microbial production has attracted significant interest recently, but its implementation is limited as per the literature studies (36). Carotenoids such as astaxanthin, lycopene, and carotene have been genetically engineered into *Escherichia coli (E. coli)*. As a result, experiments were done to develop *E. coli* cell factories that could produce crocin. *E. coli* has a distinctive genetic make-up, expands quickly under simple culture conditions, and is capable of a variety of well-known large-scale fermentation processes. The YL4 and YL5 strains are the ones that start crocin synthesis. Finally, the researchers were able to establish strains that produced crocetin and crocin-5 by integrating and optimizing the expression of the heterologous genes (37).

## Extraction of crocin by conventional method and novel techniques

Extracting bioactive components from saffron necessitates ongoing research into environmentally and economically viable extraction strategies (38, 39). Traditional extraction procedures are time-consuming and necessitate a large amount of solvent (40). As a result, novel extraction strategies for extracting bioactive components from saffron have been devised, reducing extraction time and solvent usage while improving extract extraction yield and quality (38). Several approaches have been developed to extract bioactive components from saffron with maximum extraction and purity efficiency (38). It is confirmed that, compared to traditional procedures, the targeted bioactive components can be extracted more efficiently in terms of solvent volume and extraction time by employing the right extraction method (41–43). According to El Asbahani et al. (44), the extraction method should be chosen based on the desired bioactive component, heat sensitivity, tissue complexity, etc. Conventional extraction processes (soxhlet extraction, maceration, solvent extraction vapor, or hydro-distillation) are generally non-selective, require a high volume of organic solvents, and require longer extraction times in certain cases, damage heat-sensitive bioactive chemicals (16, 45). These current extraction processes, known as “green methods,” are environmentally friendly, safer, faster, more efficient, and more precise (46). Green approaches include several extraction techniques, including membrane and emulsion liquid ultrasonic extraction, supercritical fluid extraction, enzyme-associated extraction, microwave extraction, and pulsed electric field extraction (41, 47). These approaches can efficiently extract saffron bioactive components. In general, the efficiency of extraction procedures is primarily determined by the selection of appropriate solvents, taking into account solvent-solute affinity and the employment of coextraction techniques (48). To get saffron bioactive components such as crocin, picrocrocin, and safranal, a wide range of solvents, such as water and organic solvents, and their combinations have been utilized (49). In general, fewer polar chemicals (safranal) are extracted with carbon dioxide, whereas initially, polar substances (crocin, crocetin) are extracted with organic solvents (e.g., ethanol) (50). Mohajeri et al. (51) demonstrated the extraction of crocin from saffron using molecularly imprinted polymer methods. Recent research on saffron used MAE to extract several bioactive components (picrocrocin, safranal, and crocin). The components' contents were determined spectrophotometrically at 257, 330, and 440 nm (the peak absorbance values of picrocrocin, safranal and crocin), respectively (52). The extraction and purification techniques depend on obtaining any important elements such as bioactive chemicals (crocins, crocetin, safranal, and picrocrocins) naturally found in plants. An effective bioactive extraction process should fulfill green chemistry standards such as safety, environmental friendliness, low or no contaminants, efficiency, and economics (41, 53). Saffron's key bioactive components—picrocrocin, safranal, and crocin—were extracted using subcritical water. The response surface approach was used to study the effect of extraction time (5–15 min) and temperature (105–125°C) on process efficiency. The crystallization process was used to recover complete crocin from saffron stigmas. The optimal extraction solvent was determined to be 80% ethanol. At different temperatures, the crystallization process was carried out in one and two. As a crystallization media, 80% ethanol was employed. Crocin crystals were obtained with low purity from 1st crystallization as compared to 2nd crystallization produced pure crystals at a low temperature (−5°C) quantified using UV-visible spectrophotometer and HPLC followed by Fluka product and saffron extraction using methanol (54, 55). The results showed that its purity was ~13 times greater than the Fluka products. Despite our expectations, the Fluka product was not a pure alpha-crocin sample; its chromatogram revealed five forms of crocins and an unknown impurity (54). The purity of total crystalline crocin was >97% (54). Crocin can only be split into seven fractions using this approach. The approach also necessitates time-consuming multiple treatments before pure crocin can be extracted. Table 1 shows the conventional and novel extraction methods of crocin.

**Table 1:** Extraction and separation of crocin.

**Source**	**Extraction (conventional/ novel)**	**Extraction technique**	**Conditions**	**Detection/separation**	**References**
Gardenia fruits	Conventional	50% acetone	Time: 31.4–32.2 min Temperature: −40°C	HPLC	(56)
Saffron	Conventional	Methanol–water (50:50, v/v)	Time: 24 h Temperature: 4°C	HPLC	(57)
Saffron	–	–	Time: 05 min Room temperature	HPLC	(58)
Green ovaries gardenia	Conventional	50% (v/v) ethanol	Time: 45 min Room temperature	HPLC	(59)
Gardenia fruits	–	–	Time: 28.6 min Temperature: 70.4°C	HPLC	(60)
Saffron stigma	Conventional	Water	–	Thin layer chromatography	(61)
Saffron	Conventional	Methyl alcohol	–	–	(62)
Saffron	–	–	–	Electronic tongue	(63)
Saffron	Conventional	Ethanol	Time: 4 and 3 h Temperature: 100°C	–	(64)
Saffron	Novel	Microwave-assisted extraction (MAE)	Time: 2–10 min Temperature: 100–103°C	–	(52)
Saffron	Novel	Microextraction	–	–	(65)
Saffron	Novel	Ultrasound	–	–	(66)
Saffron	Novel	Homogenate extraction	Time: 40 S Temperature: 57°C	–	(67)
Saffron	Novel	Ultrasound-assisted extraction (UAE)	–	HPLC	(68)
*Crocus sativus* L. dry stigmas	Novel	Ultrasound assisted extraction	–	HPLC	(69)
Saffron stigma	Novel	Ultrasound assisted extraction	–	UV-vis spectrophotometer	(70)
Saffron	Novel	Microwave-assisted extraction	–	–	(71)
Saffron	Novel	Supercritical carbon dioxide extraction	–	HPLC	(72)

## Food applications of crocin

Saffron is a major source of various bioactive compounds, including crocins, picrocrocin, and safranal (49). In various food products, such as bakeries and beverages, the stigma of saffron is widely used as a coloring agent and an aroma (73). Studies also show that different parts of the saffron plant are used in the development of functional food products (74). The development of functional cookies enriched with the stigma of saffron showed distinct attributes like good sensory scores, increased shelf life, and high antioxidant activities (75). Different beverages enriched with saffron petals demonstrate increased antioxidant properties after fermentation (76). Various products are available in the market encapsulated with bioactive compounds commonly found in the stigma and petals of saffron (41). The enrichment of saffron bioactive ingredients is done on a large scale. The saffron-enriched pasta products exhibit a low glycemic index due to resistant starch digestibility (77). It was also observed that the crocin-encapsulated tablet decreased the glycemic index due to reduce amylase activity in healthy people (78). Saffron carotenoids and crocin are used as coloring agents. Various food dishes are prepared with crocin, like the low kebab in Iran, pulao in India, and paella rice in Spain (Table 2).

**Table 2:** Food applications of crocin.

**Plant source**	**Food application**	**Purpose to make functional food**	**References**
–	W/O micro-emulsions	Encapsulation	(79)
Saffron	–	Nano encapsulation	(80)
Saffron	Crocin emulsion	–	(81)
Saffron	–	Nano encapsulated crocin	(82)
Saffron	Pasta	Saffron extract (crocin) seems to effect starch digestibility	(83)
–	Acidic beverages	Results show the improvement by addition of soybean soluble polysaccharide (SSPS) and avenin on the compound properties and stability of crocin in different conditions [under acidic (pH 2.5) and neutral (pH 7.0)].	(84)
Saffron	Drink	It may be given a sweet taste and aroma to the drink (wine, other alcoholic and non-alcoholic beverages)	(85)
Gardenia fruit pomace (GFP)	Steamed bread	GFP enrichment of steamed bread could retain most of the crocins and slow starch digestion that improves the appearance and nutritional quality of steamed bread.	(86)
Stigma (*Crocus sativus*)	Rye bread	The results showed a most consumer-acceptable rye bread (RB) containing saffron (S) powder (RB + S) was designed to verify its anti-diabetic properties.	(87)
Saffron	Wheat flour pasta	Saffron improved the antioxidant activity and crocin content of the pasta.	(77)

### Dairy products

The bioactive compounds of saffron are commonly used to develop various functional food products, including dairy products (88). Moreover, in dairy products, various types of cheese are developed that are enriched with saffron (89). Various cheeses, including Luneberg cheese, Pecorino allo Zafferano and Piacentinu Ennese cheese, are obtained from different animal milk sources like Austrian cow's milk, Italian sheep's milk, and Sicily sheep's milk. Enriching saffron ingredients like crocin in sheep's milk influenced physicochemical properties (90). It was evident from studies that cheese enriched with saffron showed more distinct properties than others (91). This type of cheese is yellowish in color having good elasticity and microbiologically more stable (134). A group of researchers examined the effect of saffron-rich cheese in different aspects, including physicochemical properties, sensory attributes, color, and antioxidant activities. No significant change occurred in all properties, but the saffron-enriched product's antioxidant activity and antimicrobial capacity increased (91).

### Dessert products

In dessert products, two types of dessert products, cheesecake incorporated with saffron and orange jam; the other white chocolate soup incorporated with saffron and yogurt, were evaluated in various physicochemical properties (92). A standardized level of crocin was incorporated in both types of desserts. It was concluded that the dessert incorporated with crocin had increased consumer acceptability and precise control dosage compared to dessert incorporated with saffron stigma (135). Several factors like easily water-soluble, undesirable fibers, and the small size of powder saffron do not affect the properties of the desired product. The saffron extract is considered more valuable due to its uniform color intensity and no need for preheating treatments (9).

### Cereal products

The applications of saffron in cereal products are very effective in reducing disease risk and improving the health status of the modern-age population (93). The incorporation of saffron bioactive compounds, especially crocin, in cereal products is examined in several aspects like physicochemical properties, color, texture, and sensory attributes (94). The pasta incorporated with saffron showed distinct variations in different properties, including aroma, taste, color, gumminess, hardness, chewiness, and overall acceptability (95). It was evident from the DPPH and ABTS assays that saffron-incorporated pasta showed high antioxidant properties (95). Several studies showed that the water uptake during the cooking process of pasta incorporated with a high amount of saffron is reduced due to non-soluble compounds found in saffron that are responsible for inhibiting the diffusion of water in the gluten matrix (96).

### Beverage industry

Saffron extract is widely used in the beverage industry to prepare alcoholic and non-alcoholic beverages, herbal teas, vermouth, and several bitter drinks (97). In beverages, the bitterness due to saffron extract is a limited aspect of consumers' acceptance (98). It was observed that the phenolic content in the herbal tea blend improves the bioaccessibility of crocin by reducing the crocin oxidation during the digestion process. The previous studies showed the bioavailability and bioaccessibility of saffron carotenoids in beverage industries (99).

## Effect of crocin on brain-related diseases

**Figure 2:**
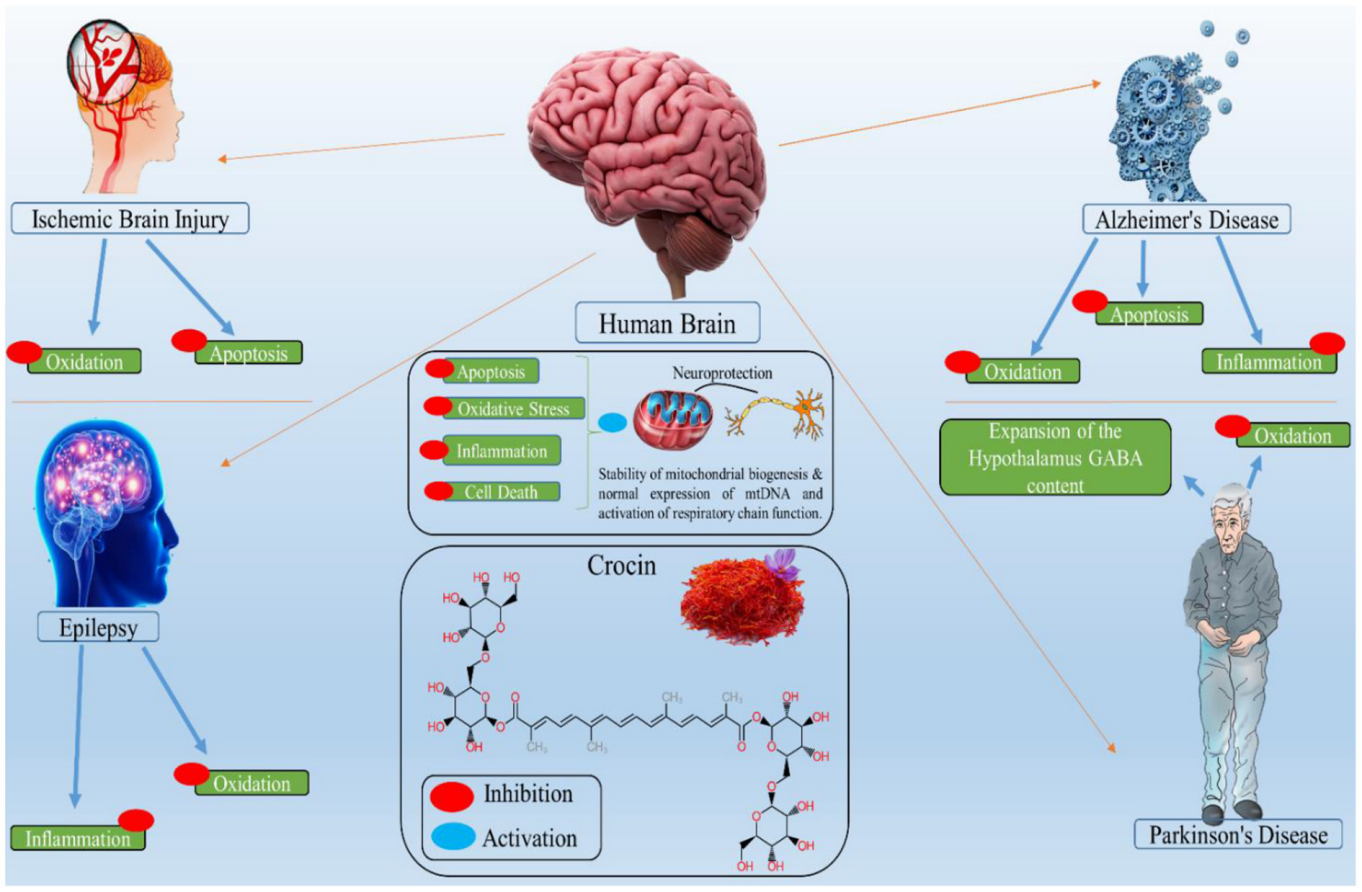
Biological activity of crocin in different brain related disorders, where the red circle shows the inhibitory property of crocin and blue circle shows the activation.

Crocin is a natural neuroprotective molecule with anti-depressant properties and potential use in treating neuropsychological problems (100). Crocin has been discovered to reduce beta-amyloid aggregation, a key step in Alzheimer's disease pathogenesis. Crocin helps those with chronic obstructive pulmonary disease and lipopolysaccharide who are depressed. Crocin can also act as an anti-inflammatory agent (101). Figure 2 presents the biological activity of crocin in different brain-related disorders. The pharmacological potential of crocin in brain-related diseases is shown in Table 3.

**Table 3:** Pharmacological potential of crocin in brain-related disease.

**Food/food part**	***In vivo/in vitro***	**Disease (brain)**	**Improvement**	**References**
*Crocus sativus L*	–	Chronic stress	Treatment with saffron extract or crocin blocked the ability of chronic stress to impair spatial learning and memory retention	(20)
Stigma	–	Alzheimer disorder, brain neurodegenerative disorder	It has universal acceptability as a phytotherapeutic drugs	(24)
Saffron	*In vivo*	Withdrawal syndrome, craving, and cognitive function	Results showed that crocin supplementation for 12 weeks to patients under MMT programs had beneficial effects on craving and withdrawal symptoms score, but did not affect the cognitive function parameters.	(102)
Saffron	*Iv vivo*	Chronic cerebral hypo perfusion	The study suggests that saffron extract and crocin improve spatial cognitive abilities following chronic cerebral hypo perfusion.	(103)
Dry stigma of the plant *Crocus sativus L*.	–	Alzheimer's disease (AD)	The results showed that crocin potential to improve learning and memory as well as protect brain cells	(104)
Saffron	*In vivo*	Brain aging (Cognitive decline and memory deficits)	This study suggested that saffron-treated mice exhibited significant improvement in learning and memory	(105)
Saffron	*In vivo*	Mild traumatic brain injury	Saffron extract and crocin provide a neuroprotective effect in a mouse model of rmTBI by decreasing oxidative stress and inflammatory responses.	(106)
Fruits of gardenia and stigmas of saffron	*In vivo*	CNS homeostasis	The finding showed that crocin and crocetin provide neuroprotection by reducing the production of various neurotoxic molecules from activated microglia.	(107)
Saffron	*In vivo*	–	The biochemical changes support the neuroprotective potential of saffron under toxicity.	
–	–	Alzheimer's disease	The result suggested that crocin may have beneficial effects in the treatment of neurodegenerative disorders (Alzheimer's disease).	(108)

### Alzheimer's

Alzheimer's is a neurodegenerative disease that causes mental capacity development and disrupts neurocognitive functions (109). Neurodegeneration, neuronal loss, and the formation of neurofibrillary tangles and Ab plaques are all signs and symptoms of this neuropathological disorder (109). Alzheimer's disease is the major cause of dementia in people over 60. Alzheimer's disease affects between 50 and 75% of patients with dementia (109). There is a lack of a logical chronological order of events in Alzheimer's disease and acceptable and effective treatment (110). The interaction of Ab protein oligomers with glial cells and neurons causes a variety of pathological and physiological abnormalities, including mitochondrial dysfunction, pro-inflammatory cascade stimulation, increased tau phosphorylation and oxidative stress, calcium metabolism deregulation, increased glycogen synthase kinase (GSK)-3b activity, cell death stimulation, and neuronal apoptosis (111, 112).

Alzheimer's disease is a difficult illness to prevent and treat because of its complex pathophysiology (113). Herbal compounds have been proposed as potential anti-Alzheimer's agents (114). Crocin, the primary component extracted from *Crocus sativus* L, has various pharmacological effects, including anti-apoptotic activity. Crocin, the primary component of *Crocus sativus* L. extract, is a yellow carotenoid with anti-inflammatory, anti-depressant, and memory-improving qualities, as well as various pharmacological activities, including anti-apoptotic capabilities (115).

Crocin's neuroprotective actions boost memory by scavenging free radicals, reducing the synthesis of peroxidized membrane lipids, and reestablishing SOD activity, reducing ROS and AGEs while lowering MDA levels and increasing GPx activity. Crocin's antioxidant effect effectively protects cerebrocortical and hippocampal neurons from ischemia, improving spatial cognitive abilities. Crocin modulated Mitogen-Activated Protein Kinase, which suppressed A concentration (MAPK) and tau phosphorylation, reducing acrolein-induced oxidative pressure. Acrolein has been linked to the development of Alzheimer's disease. In rats, acrolein at 3 mg/kg/day p.o. lowers Glutathione (GSH) levels, increases MDA, A, and Pt in the brain, and activates MAPK signaling pathways (16).

### Parkinson's disease

Parkinson's disease (PD) is the most prevalent neurological disorder. It is a progressive neurological disease that primarily affects the elderly (116). Anxiety, depression, sleep problems, and cognitive modifiability are common neuropsychiatric diseases in people with Parkinson's disease. For people with Parkinson's disease, these disturbances often create greater difficulty and distress than the disease's motor symptoms. Depression is the most frequent neuropsychiatric symptom in Parkinson's disease, with up to 50% of PD patients suffering from this psychiatric illness. The loss of nigrostriatal dopamine (DA) neurons is a characteristic symptom of Parkinson's disease (101). Development of filamentous, cytoplasmic inclusions, primary aggregations of synuclein as Lewy bodies (LB) or neurites is a pathological characteristic of PD. Fibrillization and Synuclein phosphorylation lead to the development of LB and neuron death. In Parkinson's patients, synuclein aggregates have been observed in the dorsal motor nucleus (DMN), vagus nerve, spinal sympathetic epicardium nerves, and preganglionic neurons. In 60% of PD patients with bladder hypersensitivity, urinary tract abnormalities are identified, resulting in voiding urgency, incontinence, and frequency (117).

Furthermore, it has been demonstrated that crocin treatment can inhibit AChE activation from increasing. As a result of these qualities, we chose to test crocin's neuroprotective effects against dopaminergic neuron damage and PD consequences in a model (mouse model) of this disease through 1-methyl4-phenyl-1,2,3,6-tetrahydropyridine (MPTP). Crocin also lowers depressive-like symptoms such as anxiety in adult male rats exposed to teen stress and dendritic remodeling in the PFC (prefrontal cortex). Crocin has been demonstrated as effective for disease in several investigations. Crocin also enhanced aversive memory in a Parkinson's patient model based on 6-hydroxydopamine. These findings show that crocin could be a novel contender for Parkinson's disease and depression treatment (101).

Crocin has also been shown to lower the amount of -synuclein in rats with rotenone-induced Parkinson's disease. As a result, the protective impact of crocin (10, 20, 40 mg/kg, 28 days, i.p.) on oxidative stress, malathion-induced Parkinson's disease, and inflammation in the rat striatum were examined (118).

In another animal model of disease caused due to MPTP, crocin treatment was found to reduce motor deficits and preserve dopaminergic neurons and by blocking the opening of the mitochondrial permeability transition pore (mPTP) protect against mitochondrial dysfunction (119).

### Ischemic brain injury

Hypoxic-ischemic brain damage could lead to morbidity and mortality among all age groups. One of the most common causes of infant brain damage is hypoxic-ischemic (HI) injury. In the United States, 1–4 neonatal HI brain injury occurrences occur per 1,000 live births, accounting for around one-fourth of all neonatal deaths globally. Intrauterine hypoxia related to circulation issues, such as placental abruption, placental artery clotting, and inflammatory processes, is the most common cause of neonatal hypoxic brain injury (120). Using these models, researchers have discovered numerous distinct characteristics of neonatal HI brain injury, which may be related to the nervous system's immaturity. Around birth, antioxidant enzymes (e.g., copper-zinc superoxide dismutase and glutathione peroxidase) have a restricted action in the young brain. As a result, oxidative damage induced by HI injury is more likely in the infant's brain (121).

Crocin is an active ingredient isolated from saffron with anti-inflammatory, antioxidant, and neuroprotective effects. According to a previous study, crocin pretreatment reduced cerebral edema and enhanced functional outcomes in a mouse model of traumatic brain injury. Crocin was also reported to reduce brain infarct volume in the rat ischemia-reperfusion paradigm. It is unknown whether crocin has a neuroprotective effect on HIE (122).

Crocin's therapeutic impact in reducing blood brain barrier (BBB) disruption was investigated. Twenty-four-month-old rats were administered either vehicle (controls) or crocin (10, 20, 40, or 60 mg/kg) every other day for 2 months before ischemia induction. In the presence of cerebral ischemia, Crocin preserved BBB function. In addition, the crocin-treated group had increased NADPH oxidase. The antioxidant ability of crocin was shown in these experiments to help minimize the damage induced by ischemia (123).

### Epilepsy

Epilepsy, a neurological disorder marked by recurring seizures, is frequently linked to earlier nervous system abnormalities. Epilepsy is caused by a disruption in the regulation of inflammatory cell activation and resolution in injured neuronal tissue. However, this imbalanced inflammatory regulation that contributes to epilepsy is still unknown (124). Epileptic convulsions are due to disruptions in the physiology of the brain. Abnormalities in the membrane properties of neurons, decreased inhibition of neurotransmission (by gamma-aminobutyric acid, GABA), changes in the ionic microenvironment surrounding the neuron, or increased excitatory neurotransmission (by the acidic amino acid glutamate) are all causes of epileptic seizures. In all ionotropic glutamate receptors, the intake of sodium and the outflow of potassium ions by the channels can depolarize the membrane by forming the action potential. In the resting state, Mg^++^ ions block the Ca^++^ channel in NMDA receptors, depolarizing the local membrane; channels change to permeable for calcium ions with the shifts of Mg^++^. In high neuronal activation (e.g., status epilepticus), Ca^++^ inflow can cause cell depolarization to play a part in Ca^++^-mediated neuronal damage, leading to cell death (125).

Because epilepsy involves complicated neural pathogenic pathways, multi-targeted pharmacological treatments have been proposed for its complete care. It is been studied extensively in animal models of neurological disorders, including depression, epilepsy, anxiety, and memory. Crocin's efficacy in neurological diseases characterized by aberrant central excitatory and inhibitory nature shows that it interacts with various neuronal pathophysiological pathways (126).

Crocin has an unsettling effect on the cell reinforcement framework, resulting in increased ROS production and subsequent ROS-induced mitochondrial malfunction, frequently found following seizures and throughout epileptogenesis. Its antioxidant properties also aid crocin's antiepileptic properties. Antiepileptic medicines improve GABA-mediated inhibition and raise GABA levels in the brain (16). Crocin infusions in penicillin-activated epileptiform action in rats resulted in antiepileptic effects. Crocin's antiepileptic effect is due to its ability to increase the tone of inhibitory neurotransmitters by increasing the working of GABA (A)-benzodiazepine. Crocin stimulates glutamic acid decarboxylase, an important enzyme for GABA synthesis, greatly increasing the hypothalamic GABA concentration in rats (127).

## Conclusions

The present study is deliberate to measure the effects of crocin extract in functional foods and its pharmacological properties against various disorders. Crocin is a biologically active substance in the stigma of saffron and fruit of guardian. These bioactive substance can be extracted utilizing conventional (solvent extraction, soxhlet extraction, vapor or hydro distillation, and maceration), and novel techniques (supercritical fluid extraction, microwave-assisted extraction, ultrasound-assisted extraction, pulsed electric field extraction, emulsion liquid membrane extraction and enzyme-associated extraction). In various food products, such as bakeries and beverages, the stigma of saffron is widely used as a coloring agent and an aroma. The antioxidant profile of crocin, may inhibit the oxidation process in different foods. The development of baking products, beverages, dairy products, dessert and cereal products are enriched with the stigma of saffron showed distinct attributes (sensory scores, good shelf life, antioxidant activity). These products having saffron in them have very effective in reducing disease risk and improving the health status of the population. Pharmacologically, crocin may be helpful in different brain-related disorders, including Alzheimer's, Parkinson's, Ischemic brain injury, and Epilepsy.

## Author contributions

AAl, SK, WK, and MM: conceptualization. AAl, SK, WK, ZM, AAz, and MA: writing-original draft preparation. RA, MT, LY, HQ, SR, HS, and MM: writing-review and editing. LY, HQ, and MM: supervision. All authors have read and agreed to the published version of the manuscript.
